# Exploring the Genome of a Butyric Acid Producer, *Clostridium butyricum* INCQS635

**DOI:** 10.1128/genomeA.01169-14

**Published:** 2014-11-20

**Authors:** Thiago Bruce, Fernanda Gomes Leite, Diogo Antonio Tschoeke, Milene Miranda, Nei Pereira, Rogério Valle, Cristiane C. Thompson, Fabiano L. Thompson

**Affiliations:** a Faculdade de Tecnologia e Ciências, Grupo de Biotecnologia Ambiental, Salvador, BA, Brazil; b Laboratório de Microbiologia, Instituto de Biologia, Universidade Federal do Rio de Janeiro (UFRJ), Rio de Janeiro, RJ, Brazil; c Laboratório de Bioprocessos, UFRJ, Rio de Janeiro, RJ, Brazil; d Sage-Coppe, UFRJ, Rio de Janeiro, RJ, Brazil

## Abstract

The draft genome sequence of *Clostridium butyricum* INCQS635 was obtained by means of ion sequencing. The genome provides further insight into the genetic repertoire involved with metabolic pathways related to the fermentation of different compounds and organic solvents synthesis (i.e., butyric acid) with biofuel applications.

## GENOME ANNOUNCEMENT

The genus *Clostridium* (phylum *Firmicutes*) comprises 200 bacterial species with the well-established ability to consume hexose and pentose sugar monomers, which are metabolized through fermentation and used to produce organic solvents on an industrial scale (1). Butyric acid is commonly produced by certain types of *Clostridium* that can be used to produce ethyl butyrate and butyl butyrate under anaerobic conditions. For instance, *C. butyricum* can convert carbohydrate polymers to butyric acid. There is great interest in the use of these compounds as biofuel precursors (2). The genomic sequence of *C. butyricum* is a tool to improve industrial production of butyric acid. The ability to convert complex sugars in the pretreatment of biomass waste used as raw material for biofuel production by means of microbial enzymes represents a promising alternative to thermo-chemical processes currently applied in the production of second generation bioethanol (3).

The genomic sequence of *C. butyricum* INCQS365 was determined by means of ion sequencing. *C. butyricum* INCQS635 DNA was extracted using the Pitcher method (4). The sequencing was performed as described by Quail et al. (5). The Ion PGM 200 sequencing kit was used for all sequencing reactions, following the recommended protocol. The reads obtained were assembled using *de novo* assembly on the Ion Torrent platform based on MIRA software (6), followed by a second assembly using CAP3 software (7).

The genome sequence was annotated by means of the subsystems approach through the RAST server (8) and the identification of conserved domains present in genes encoding for carbohydrate-active enzymes (CAZYmes) were identified using the CAZYmes analysis toolkit (e-value 10^−5^) (9).

The assembly of *C. butyricum* INCQS635 genome sequencing generated 231 contigs and a total size of 4,407,025 bp (14× coverage) and a G+C content of 28.51%. The *N*_50_ contig length was 31,600 bp. A total of 87 RNAs and 5,771 protein coding sequences were identified. From this, 2,626 were classified into 376 subsystems. The carbohydrate subsystems corresponded to 18% of the genomic sequences of *C. butyricum* INCQS635 from which 10% of the sequences were involved in fermentation processes. The search for glycosyl hydrolases (GH) identified 182 (3.1% or 182/5,771) coding sequences that represent CAZYmes in *C. butyricum* INCQS365. Most enzymes were classified in the large GH family, reaching 58% of all CAZYmes. GH13 (comprising, e.g., pullulanases and α-amylases) and GH23 (comprising lyzozymes and chitinases) families were the most abundant, with 16% and 11%, respectively, of all identified sequences. *C. butyricum* INCQS365 shows promise for the production of organic acids (e.g., butyric acid) by means of the hydrolyses of starch and/or chitin-rich biomasses.

### Nucleotide sequence accession numbers.

This whole-genome shotgun project has been deposited at DDBJ/EMBL/GenBank under the accession no. JRMA00000000. The version described in this paper is version JRMA01000000.
